# The temporal dynamics of visual crowding in letter recognition: Modulating crowding with alternating flicker presentations

**DOI:** 10.1167/jov.23.10.18

**Published:** 2023-09-28

**Authors:** Andrew E. Silva, Rebecca Lehmann, Niki Perikleous, Benjamin Thompson

**Affiliations:** 1 School of Optometry and Vision Science, University of Waterloo, Waterloo, Ontario, Canada; 2 Aalen University, Optics and Mechatronics, Aalen, Baden-Wuerttemberg, Germany; 3 Aalen University, Optics and Mechatronics, Aalen, Baden-Wuerttemberg, Germany; 4 School of Optometry and Vision Science, University of Waterloo, Waterloo, Ontario, Canada; 5 Centre for Eye and Vision Research, Hong Kong, SAR China; 6 Liggins Institute, University of Auckland, Auckland, New Zealand

**Keywords:** crowding, temporal crowding, peripheral vision, letter recognition

## Abstract

Visual crowding reduces the visibility of a peripherally presented group of stimuli. This is especially challenging for peripheral reading because adjacent letters or characters perceptually crowd one another. We investigated the temporal course of spatial visual crowding by sequentially alternating the visibility of the target and flanking letters within a trigram letter stimulus presented 9° below fixation. We found that alternation rates of roughly 3 Hz released half of the total effect of crowding, whereas 10 Hz alternation rates elicited near-crowded performance. Furthermore, we found a robust performance asymmetry whereby presenting the target first elicited better performance than presenting the flankers first, an effect resembling forward masking. These results held for conditions of high, medium, and low spatial crowding. Future work will determine whether the alternation rates found in the current study can improve peripheral reading.

## Introduction

Identifying objects and reading text is markedly more difficult when using peripheral vision compared to central vision. Not only does peripheral viewing suffer from degraded visual acuity, but it is also prone to visual crowding. For example, a letter presented to peripheral vision is easier to identify when presented alone than when presented as the central letter of a trigram (Levi, 2011; Whitney & Levi, 2011). The flanking letters perceptually crowd the central letter, resulting in worse letter recognition. Crowding may be especially relevant for patients with central vision loss who must read with their relatively intact peripheral vision. As a result, several methods to reduce visual crowding for letter and word stimuli are currently under investigation in normal and visually-impaired observers, including the use of noninvasive brain stimulation and prolonged reading training (Chung, 2011; Chung, Legge, & Cheung, 2004; Contemori, Trotter, Cottereau, & Maniglia, 2019; Donkor et al., 2021; Silva et al., 2022). However, the properties of the visual stimulus itself can also be manipulated.

Visual crowding is strongest when the target and flanking stimuli are close together, exhibit similar features, and are arranged along the axis of fixation (Bernard & Chung, 2011; Toet & Levi, 1992). Therefore crowding can be attenuated by increasing the separation between objects and varying color or contrast polarity between the targets and crowders (Kennedy & Whitaker, 2010; Levi, 2011; Scolari, Kohnen, Barton, & Awh, 2007; Whitney & Levi, 2011). Unfortunately, although these manipulations reduce crowding, they do not necessarily improve peripheral reading performance. Reading fluency is dependent on the recognition of the holistic word shape, and therefore manipulations that disrupt word shape such as contrast polarity shifts or increased letter spacing may negatively impact reading fluency even while alleviating between-letter crowding (Chung, 2002; Chung & Mansfield, 2009).

Prior temporal crowding experiments in which each stimulus item was presented one letter at a time have demonstrated a temporal crowding effect that wanes with increasing interstimulus interval (Chung, 2016; Chung & Patel, 2022; Tkacz-Domb & Yeshurun, 2021; Yeshurun, Rashal, & Tkacz-Domb, 2015; Yu et al., 2020). In these studies, the components of each trigram were generally presented with a fixed exposure duration, such that longer interstimulus intervals corresponded to longer trial durations. Temporal manipulations have also been adapted for reading and word recognition tasks. For example, sentences presented one word at a time, sometimes in the same spatial location to minimize eye movements, can impart some reading benefits (Rubin & Turano, 1992; Wallis, Yang, & Anderson, 2018). Additionally, Haberthy and Yu (2016) presented individual words to participants 10° below fixation, finding that small text was read more quickly if the letters composing the word were presented one at a time (moving window condition), or if a single obscured letter swept across the word (moving scotoma condition). However, the maximum reading speed achieved by participants suffered as a result of these temporal manipulations.

Although visual crowding reduction using temporal manipulations has so far resulted in some limited reading improvements (Haberthy & Yu, 2016), its overall efficacy may be complicated by additional unintuitive perceptual effects. For example, “distractor preview” is a well-described asymmetry in the strength of temporal visual crowding; crowded stimuli in which the crowders appear before the target elicit less crowding than if the crowders appear after the target (Chung & Patel, 2022; Scolari et al., 2007; Soo, Chakravarthi, & Andersen, 2018). Distractor preview may overlap with temporal forward and backward masking, although they often operate on different temporal scales than distractor preview (Chung, 2016; Huckauf & Heller, 2004; Tkacz-Domb & Yeshurun, 2021). Forward masking occurs when two objects are presented sequentially, and the presentation of the first object inhibits the perception of the second object. In contrast, backward masking occurs when sensitivity to the first object is reduced by the presentation of the second object.

Moreover, experiments where individual transient onset and offset “blinks” were added to either the target or the crowders demonstrated an asymmetry whereby a blinking target released crowding, but blinking crowders maintained crowding (Greenwood, Sayim, & Cavanagh, 2014). Consistent with distractor preview, the crowding reduction was primarily driven by the delayed onset of the target within the blink (Greenwood et al., 2014). Similar effects of transient stimulus onset and offset periods have also been demonstrated in other areas, such as in change detection (Phillips & Singer, 1974).

In the current study, we endeavored to better understand the consequences of applying a temporal manipulation to a spatially crowded sequence of letters. We presented trigram letter stimuli and asked participants to identify the middle target letter. Critically, we alternated the presentation of the flankers and the target repeatedly during a trial, investigating the relationship between the alternation rate and target recognition. Therefore the total stimulus duration remained constant. A slow 1 Hz alternation of the target and flankers, with each visible for 500 ms, was hypothesized to elicit performance akin to an uncrowded presentation. Performance was expected to decrease with faster alternation rates until reaching levels elicited by standard crowded trigram displays. Although this novel task is distinct from true reading, it allows for the investigation of the temporal course of the strength of visual crowding. In addition, it allows a direct investigation of the effects of backward and forward masking with the target initially presented either before or after the flankers—effects that would be impossible to measure with a word reading task.

We also tested three unique crowding configurations to determine whether the temporal course of spatial visual crowding differed between high, medium, and low crowding displays. Low spatial crowding stimuli were created by introducing contrast polarity differences between the target and flankers (Chung & Mansfield, 2009), and high spatial crowding stimuli were created by orienting the flankers perpendicular to the axis of fixation (Toet & Levi, 1992). Although these additional spatial manipulations are not relevant for reading, it is important to investigate stimuli with varying levels of baseline spatial crowding to understand more fully how temporal manipulations impact the visibility of various crowded visual scenes. Ultimately, our aim was to better understand the ways in which temporal manipulations influence letter recognition in crowded letter presentations as a first step toward determining the feasibility of temporal manipulations to improve peripheral reading.

## Methods

### Participants

Twenty-seven participants with normal or corrected-to-normal vision, between 20 to 35 years old, were recruited across three different between-subject stimulus configurations. Ten participants (two male, eight female) viewed horizontally oriented stimuli, 10 other participants (four male, six female) viewed vertically oriented stimuli, and the final seven (one male, six female) viewed horizontally oriented stimuli with reversing contrast polarity. All participants reported normal or corrected-to-normal vision and no known eye disease. For participating in the single one-hour experimental session, each subject was remunerated Can$20. The study was approved by the Ethics Board of the University of Waterloo, and the protocols adhered to the tenants of the Declaration of Helsinki.

### Apparatus, stimulus, and procedure

All stimuli were created using PsychoPy-2022.1.3 (Peirce et al., 2019), running on a desktop PC (model: Dell XPS 8930, processor: Intel Core i7-9700 CPU @ 3.00 GHz). The stimuli were presented on a 24-inch VIEWPixx LCD monitor with 1920 × 1080 pixel resolution running at a 60 Hz refresh rate.

On each trial, 3 SLOAN optotype letters of height 0.8° were presented 9° below fixation. The presented letters were randomly selected without replacement from a pool of nine options: D, H, K, N, O, R, S, V, and Z. Participants verbally identified the central target letter. No corrective feedback was given, and participant responses were not restricted to the nine possible correct answers. The viewing distance was 72 cm, and a headrest was used. Participants fixated on a bright cross of diameter 0.4°. A Tobii 4C eyetracker was used to verify fixation.

**Figure 1.**
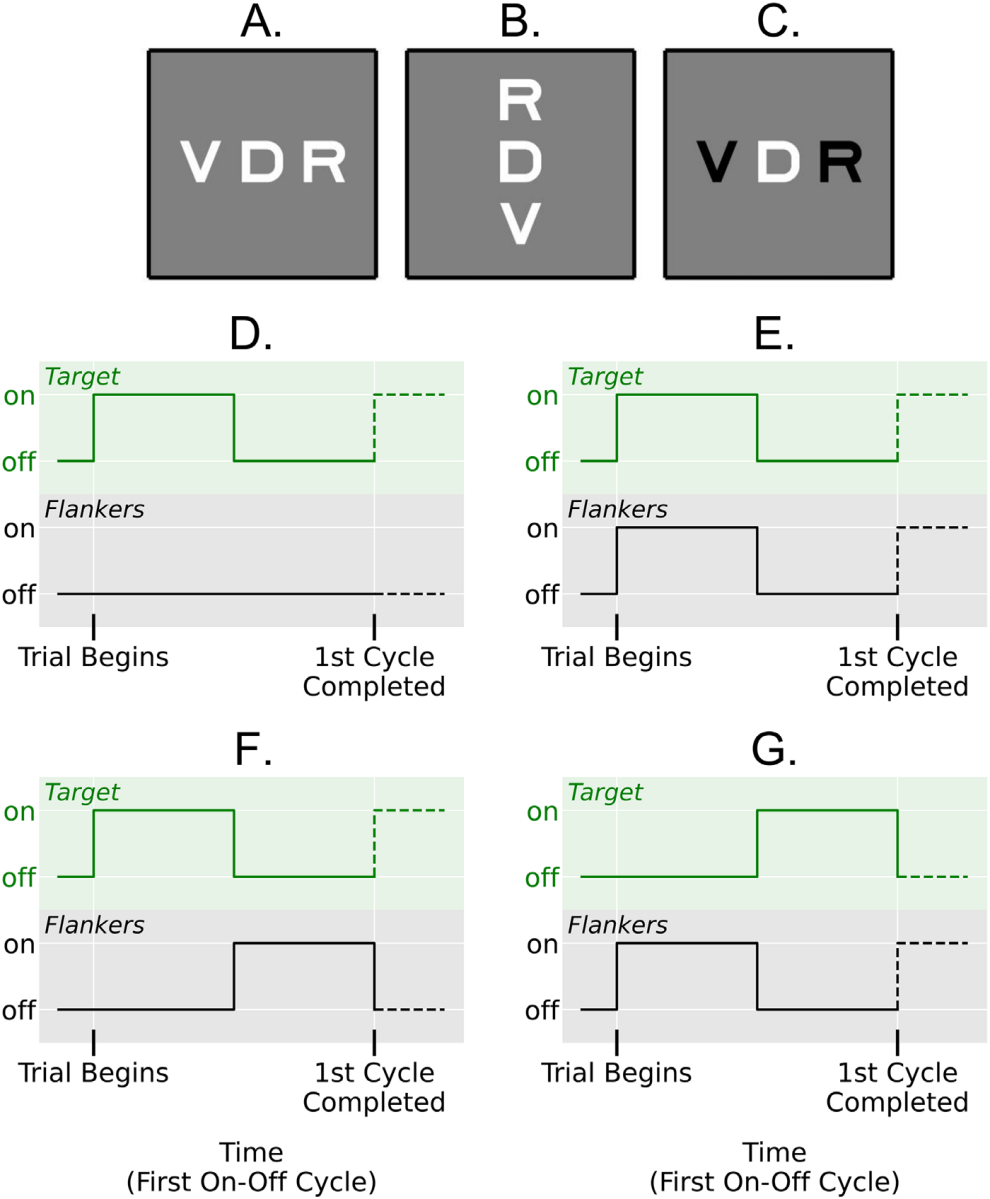
Diagrams of the three between-subject stimulus configurations (A–C) and the first cycle of the target and flanker onsets/offsets of the four within-subject temporal conditions (D–G). Participants fixated a central cross, while three random SLOAN optotypes were presented 9° below fixation., and the central target was verbally identified. (A) Horizontally oriented stimulus configuration. (B) Vertically oriented stimulus configuration. (C) Contrast-polarity stimulus configuration. Each participant only saw one stimulus configuration. (D) Uncrowded condition time course. (E) Crowded condition time course. (F) Alternating target-first time course. (G) Alternating flankers-first time course. Static (nonperiodic) crowded and uncrowded stimuli were also presented in which all letters were visible for the entire one-second stimulus duration. See Supplemental Material Videos S1, S2, S3 and S4 for example movies of each within-subject temporal condition flickering at 2 Hz.

Three different crowding strength stimulus configurations were tested. First, the horizontal stimulus configuration featured three horizontally oriented letters and was expected to elicit middle levels of spatial crowding. Second, the vertical stimulus configuration featured three vertically oriented letters and was expected to elicit stronger levels of spatial crowding because it was oriented along the axis of fixation (Toet & Levi, 1992). In both the horizontal and vertical configurations, the three letters were bright (110 cd/ m^2^) against a dark background (6 cd/m^2^). The midpoints of adjacent letters were spaced 1° apart, leaving a gap of 0.2° between letters. Finally, the contrast polarity stimulus configuration featured three horizontally oriented letters, with the target and flankers exhibiting opposite contrast polarities to elicit the weakest levels of visual crowding (Chung & Mansfield, 2009). Half of the contrast-polarity trials featured a bright target and dark flankers, and the other half featured a dark target and bright flankers. The screen was psychophysically gamma corrected using the built-in PsychoPy calibration script during the contrast polarity experiment, and the background was set to mid-gray. See Figure 1 A through C for diagrams of all three stimulus configurations.

Four within-subject temporal conditions were run per stimulus configuration:

1. In the uncrowded condition, the central target was presented alone. Three levels of the temporal manipulation were examined to examine the effect of onset and offset transients: On any given trial, the target was visible for the entire one second presentation time, or was flickered on and off at 1 Hz (60 frames per on-off cycle), or was flickered on and off at 10 Hz (six frames per on-off cycle).
2. In the crowded condition, the target and flankers were both presented simultaneously. As in the uncrowded condition, all three letters were visible for the entire one second presentation time, were flickered on and off synchronously at 1 Hz, or were flickered on and off synchronously at 10 Hz on any given trial.
3. In the alternating target-first condition, the target and flankers alternated in visibility. The rate of alternation was varied along six levels and was one of the following on any given trial: 1 Hz (60 frames per cycle, target and flankers alternated every 500ms), 2 Hz (30 frames per cycle, target and flankers alternated every 250 ms), 3 Hz (20 frames per cycle, target and flankers alternated every 167 ms), 4.3 Hz (14 frames per cycle, target and flankers alternated every 117 ms), 6 Hz (10 frames per cycle, target and flankers alternated every 83 ms), or 10 Hz (6 frames per cycle, target and flankers alternated every 50 ms). Critically, the target was always presented first during alternating target-first trials. Note that due to the 60 Hz display and the 1 second stimulus duration, there was an unavoidable but slight asymmetry in the presentation time of target and flankers during trials with a 4.3 Hz alternation rate. Potentially impacted analyses were run with and without this condition.
4. The alternating flankers-first condition was identical to the target-first condition, except that the flankers were always presented first on each trial.

Figures 1D through G presents diagrams of the onset and offset times within one cycle of all four temporal conditions. In addition, please see Supplementary Material for example movies. The onset and offsets of each stimulus followed a periodic square-wave temporal profile in which the letters were either invisible or maximally visible, and the target and flankers were never visible simultaneously in the alternating target-first and flankers-first conditions. All four within-subject conditions were randomly interleaved. Before formal data collection began, participants were given 12 practice trials for task familiarization. The main task contained 40 trials of each combination of condition and alternation/flicker rate, totaling 720 trials. A subsequent trial was presented 500 ms after the response to the previous trial was registered.

## Results

### Gaze positions

All trials where fixation deviated by 1.5° were removed from further analysis. Less than 10% of trials were excluded for 17/27 participants. Less than 15% of trials were excluded for 24/27 participants. No more than 27% of trials were excluded for any individual participant. Alternate fixation deviation cut-off values were examined, and the results of the following analyses did not meaningfully change with 1° and 2° cutoffs, nor did the results meaningfully change when using each participant's entire dataset.

### Strength of static crowding

All statistical tests were carried out using JASP v.0.16.4 (JASP Team, 2022). First, we confirmed whether the three stimulus configurations produced the expected pattern of spatial crowding strength, with the least crowding expected in the contrast polarity experiment, middle levels of crowding expected in the horizontal, and the highest level expected in the vertical experiment. The strength of crowding was calculated by subtracting the crowded percent correct accuracy from the uncrowded accuracy. A between-subjects one-way analysis of variance (ANOVA) showed a significant effect of stimulus configuration, *F*(2, 24) = 28.0, *p* < 0.0001. Planned repeated contrasts found that the contrast polarity crowding effect (23% ± 7) was indeed significantly smaller than the horizontal crowding effect (52% ± 4%), *t*(24) = 4.3, *p* = 0.0003. Likewise, the horizontal crowding effect was significantly smaller than the vertical crowding effect (74% ± 4%), *t*(24) = 3.5, *p =* 0.0017.

### Intrinsic flicker effects

**Figure 2.**
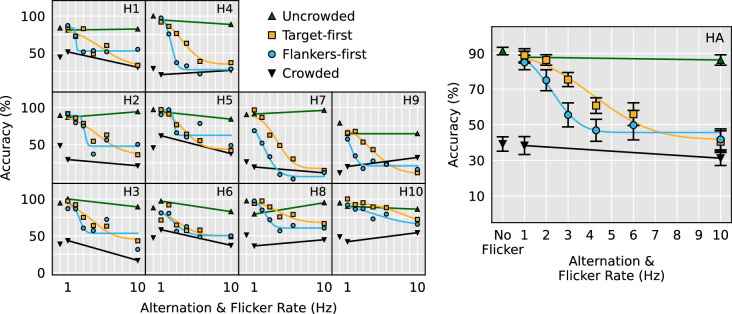
Horizontal stimulus configuration data as a function of alternation and flicker rate. Panels H1 to H10 show individual subject data and psychometric fits. Panel HA shows overall results and the psychometric fit for the averaged performance across all participants. Here and in all future figures, the error bars are ± standard error of the mean.

To examine whether the onset and offsets caused by the temporal manipulation influenced our measure of crowding within each stimulus configuration, the crowded and uncrowded data from the horizontal, vertical, and contrast polarity configurations were submitted to separate 2 (Crowding condition: crowded and uncrowded) × 3 (Flicker rate: no flicker, 1 Hz flicker, and 10 Hz flicker) ANOVAs. In all three stimulus configurations, a significant effect of crowding condition was found, horizontal: *F*(1, 9) = 184.2, *p* < 0.0001, vertical: *F*(1, 9) = 436.4, *p* <0.0001, contrast polarity: *F*(1, 6) = 15.1, *p* = 0.0081. The contrast polarity configuration ANOVA revealed a significant main effect of flicker, *F*(2, 12) = 4.3, *p* = 0.0388, but no other main effects of flicker or interactions were significant. Figures 2 through 4 present averaged and individual subject data from the horizontal, vertical, and contrast polarity configurations, respectively.

### Effects of fast and slow flicker

To examine whether alternating presentations of the flankers and targets sensibly modulated the strength of visual crowding, the 1 Hz and 10 Hz flicker data from each stimulus configuration were submitted to separate 2 (Flicker speed: 1 Hz and 10 Hz) × 4 stimulus condition (crowded, uncrowded, target-first, flankers-first) repeated measures ANOVAs. We found a significant interaction between stimulus condition and flicker speed in all three stimulus configurations, horizontal: *F*(3, 27) = 36.2, *p* < 0.0001, vertical: *F*(3, 27) = 28.4, *p* < 0.0001, contrast polarity: *F*(3, 18) = 9.1, *p* = 0.0007. Simple main effects revealed that although no significant effect of flicker speed was elicited by crowded and uncrowded stimuli, the effect of flicker speed was highly significant in the alternating target-first and flankers-first conditions. This pattern of results held for all three stimulus configurations (Table 1). Panels HA, VA, and CA in Figures 2 through 4, respectively, demonstrate that the significant interaction is due to the alternating stimulus conditions eliciting near-uncrowded performance during slow 1 Hz flicker and near-crowded performance during fast 10 Hz flicker.

**Table 1.** Simple main effects for the interaction between stimulus type and flicker rate.

	Accuracy % (1 Hz-10 Hz)	SEM	F	DOF	p
Horizontal configuration				(1, 9)	
Uncrowded	1.59	2.92	0.30		0.600
Target-first	48.49	5.65	73.60		<0.001^*^
Flankers-first	43.21	4.23	104.41		<0.001^*^
Crowded	7.10	4.98	2.04		0.187
Vertical configuration				(1, 9)	
Uncrowded	3.67	2.84	1.67		0.229
Target-first	52.83	6.47	66.69		<0.001^*^
Flankers-first	43.62	6.16	50.11		<0.001^*^
Crowded	−0.52	3.38	0.02		0.880
Contrast configuration				(1, 7)	
Uncrowded	1.26	1.82	0.48		0.515
Target-first	29.73	8.57	12.03		0.013^*^
Flankers-first	23.26	5.89	15.57		0.008^*^
Crowded	11.40	4.84	5.55		0.057

*Notes*: % = percent; SEM = standard error of the mean; F = statistical distribution value; DOF = degrees of freedom; p = probability under null distribution.

* Statistical significance at the 0.05 level.

### Target-first versus flankers-first

To investigate how different alternation rates differentially modulated performance, cumulative Gaussian psychometric functions were fit to the target-first and flankers-first data separately for each participant using the curve_fit function of the Python SciPy module. At a slow enough alternation rate, both alternating stimuli would appear functionally like static uncrowded presentations. Therefore each participant's static uncrowded performance was taken as the ceiling of the psychometric function. The floor of the psychometric function was independently fitted for each participant and stimulus condition. The alternation rate eliciting performance halfway between the two extremes, represented by the µ parameter of the fitted Gaussian, was taken to quantify the impact of alternation rate on performance. Larger µ values indicate better tolerance of faster alternation rates before reaching crowded performance levels.

**Figure 3.**
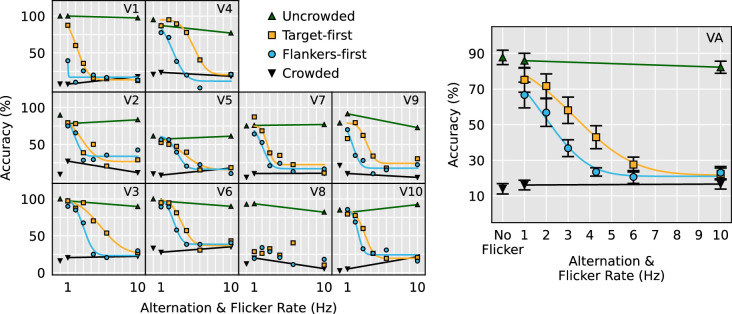
Vertical stimulus configuration data as a function of alternation and flicker rate. Panels V1 to V10 show individual subject data and psychometric fits. Note that participant V8’s data could not be fitted, so subsequent statistical analysis omitted their data. Panel VA shows overall results and the psychometric fit for the averaged performance across all participants.

To directly compare the effect of the two alternating flicker conditions across all three stimulus configurations, a 3 (Stimulus configuration, between-subjects factor: horizontal, vertical, contrast polarity) × 2 (Temporal condition, within-subjects factor: target-first and flankers-first) mixed ANOVA was performed on the fitted µ values. Only the effect of temporal condition was significant, *F*(1, 23) = 47.1, *p* < 0.0001 (target-first µ: 4.0 Hz, flankers-first µ: 2.5 Hz). It should be noted that participant V8 performed equally poorly to all trial types except uncrowded, rendering psychometric functions impossible to fit (Figure 3^4^). Therefore their data were not submitted to this analysis. Figure 5 demonstrates that faster alternation flicker rates are tolerated when the target appears first in all three stimulus configurations.

**Figure 4.**
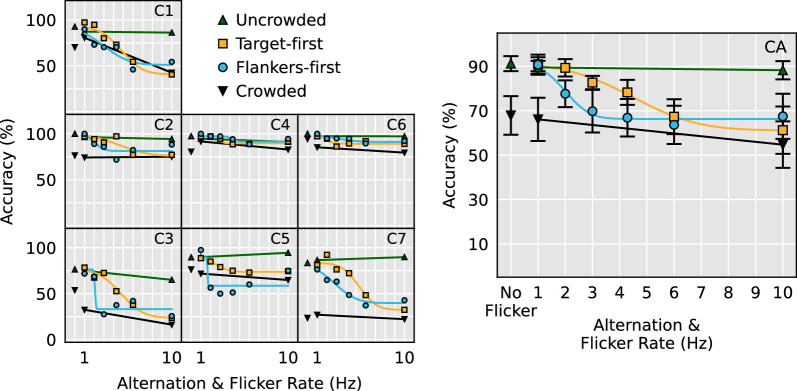
Contrast polarity stimulus configuration data as a function of alternation and flicker rate. Panels C1 to C7 show individual subject data and psychometric fits. Panel CA shows overall results and the psychometric fit for the averaged performance across all participants.

**Figure 5.**
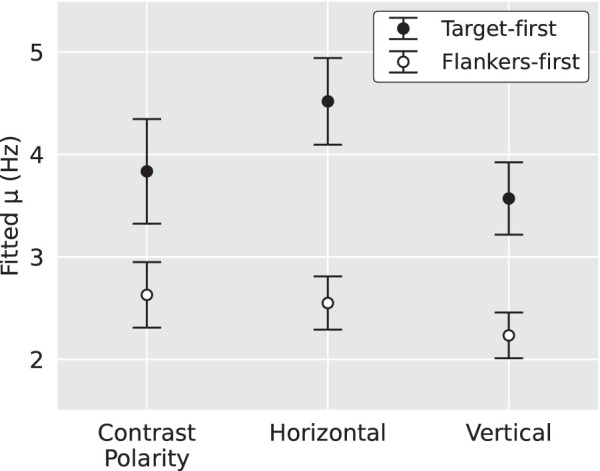
Average fitted µ values across all stimulus configurations and all alternating temporal conditions. A larger µ suggests a greater tolerance for faster alternation rates.

Because the 4.3 Hz alternation rate trials were imbalanced between flanker and target onsets, this analysis was rerun with new fitted µ values omitting the 4.3 Hz alternation rate trials. The pattern of results was unchanged, with statistical significance only for the effect of temporal condition *F*(1, 23) = 17.6, *p* = 0.0003 (target-first µ: 3.9 Hz, flankers-first µ: 2.6 Hz).

## Discussion

We examined the temporal extent of spatial visual crowding using trigram stimuli under conditions of low (contrast polarity), medium (horizontal), and high (vertical) crowding. We observed a perceptual benefit only when stimuli alternated at relatively slow rates. Also, onset and offset transients did not contribute a noticeable performance reduction during high and medium crowding trials but did so during low crowding trials. Furthermore, although the absolute crowding strength differed as expected across our low, medium, and high crowding stimulus configurations, we found similar temporal dynamics of spatial visual crowding across all three, suggesting that stimulus spatial characteristics may not determine the effectiveness of temporal manipulations for alleviating visual crowding.

In the current study, participants recognized the middle target letter within a trigram stimulus. This task is subject to strong visual crowding, particularly when the trigrams are arranged along the fixation axis (Toet & Levi, 1992), a finding replicated in our current results. We found that a slow enough temporal alternation rate (roughly 3 Hz) between target and flankers imparts a relatively large performance benefit. In contrast, effective word reading involves additional processes not tested here. English reading requires an integration of multiple letters, and thus the overall shape of the word influences reading fluency (Chung, 2002; Chung & Mansfield, 2009). By considering trigram stimuli and eliminating the effects of word shape, we specifically examined the perceptual consequences of temporal manipulations on letter recognition. Our results represent a “best-case” scenario in which detrimental effects on word shape are eliminated.

Performance was particularly poor when the flankers appeared before the targets, suggesting that this manipulation was especially impacted by the effects of forward masking. Therefore separating the presentation of different letters within a word may disproportionately impede recognition of the trailing letters within a word, potentially limiting benefits to reading. Nevertheless, our results found that even in the conditions eliciting the strongest forward masking, an alternation rate of 2.5 Hz (2.5 cycles of alternation between target and flankers per second), corresponding to a 200 ms letter presentation time, alleviated at least half of the total crowding strength during flankers-first trials.

Temporal manipulations are of interest for text recognition because they represent a potential crowding-reduction mechanism independent of letter appearance, particularly if the letter presentation times are fast enough to maintain the perceptual appearance of the word. Interestingly, the current results suggest that the optimal presentation time is markedly slower than that reported in previous temporal reading manipulations. For example, the entire temporally-modulated stimulus from Haberthy and Yu (2016) was presented within a 176 ms period. Future work may determine whether the slower alternation rate found in the current study may provide a greater reading benefit.

The current forward masking results run contrary to previous studies of temporal crowding. Studies using trigrams with single sequential presentations of target and flankers have largely found a perceptual benefit when the target follows the flankers, a so-called “distractor preview” stimulus (Scolari et al., 2007; Soo et al., 2018). The effect of distractor preview has been previously explained as an attentional prioritization of newer images. However, bottom-up inhibitory interactions between transient and sustained processing channels activated differentially by the target and flankers have been recently proposed (Chung & Patel, 2022). Consistent with this, earlier work postulated that when the target “blinks” or appears after the flankers, it isolates the target within the brain's transient processing channel and allows accurate identification, explaining the distractor preview effect (Greenwood et al., 2014).

The current investigation differed from previous work in that the target and flankers alternated repeatedly. Multiple presentations of both target and flankers occurred within the same trial in all alternation conditions above 1 Hz, and the alternation conditions never concurrently presented the target and flankers. Therefore the transient processing channel would be expected to represent target and flankers in alternation, with each isolated for a short period of time. It is unclear why this resulted in better performance during target-first trials rather than equal performance between the two alternation conditions. However, we note that target-first trials received a short but uninterrupted presentation of the target at the beginning of the trial. This initial presentation may have allowed a stronger representation of the target to form, resulting in better performance during target-first trials. Also, the flankers-first trials may have been affected by poorer attentional deployment to the target's location, perhaps resulting from increased stimulus position uncertainty due to the initial onset of the flankers. Interestingly, we did not find effects consistent with distractor preview even in the 1 Hz alternation rate condition, suggesting the perceptual consequences of different temporal manipulations are highly dependent on the specific properties of the tested stimulus.

## Conclusions

In the current study, we examined the temporal dynamics of visual crowding in trigram stimuli with alternating presentations of targets and flankers. Uniquely, the alternation rate was varied, and there was no blank interstimulus period, allowing the presentation time to remain constant. We found that relatively slow alternation rates were required to modulate the strength of spatial visual crowding; roughly 3 Hz (167 ms individual presentation time) between neighboring letters released half of the total crowding in the stimulus. Moreover, a stark performance asymmetry was found in the direction opposite of the traditional distractor preview effect. Slow baseline alternation rates and asymmetrical effects of forward and backward masking, regardless of the direction, may undermine the global integration of presented text. Therefore it will be interesting for future work to determine whether different stimulus characteristics or procedures can remove the asymmetry reported here and enable faster alternation rates with the potential to better improve peripheral reading.

## Supplementary Material

Supplement 1

Supplement 2

Supplement 3

Supplement 4
